# Advancing hypertension management in Asia 2021, beyond COVID‐19

**DOI:** 10.1111/jch.14211

**Published:** 2021-02-14

**Authors:** Aletta E. Schutte

**Affiliations:** ^1^ School of Population Health University of New South Wales Sydney NSW Australia; ^2^ The George Institute for Global Health Sydney NSW Australia; ^3^ Hypertension in Africa Research Team, MRC Unit for Hypertension and Cardiovascular Disease Potchefstroom South Africa

When entering 2021—independent of which country or continent we live in—we are overwhelmed in the scientific literature, general media, and our personal lives on the impact that the coronavirus disease (COVID‐19) is having on each of us. It has restricted almost every facet of our lives, leaving a perception that COVID‐19 is disproportionately contributing to deaths. In January 2021, there have already been almost 2.1 million COVID‐19‐related deaths reported globally over the past year. Yet, based on the figures of the Global Burden of Disease study, from January to December 2019 a total of 10.8 million hypertension‐related deaths occurred.^1^ The situation is complex. Hypertension is the most common comorbidity in hospitalized COVID‐19 patients—as reported in 5700 patients in New York, where 56.6% had hypertension.^2^ While millions are spent on vaccinating and addressing COVID‐19, more than ever we need much greater emphasis on preventing, treating, and controlling hypertension, which is remaining the “silent killer”.

The timing is therefore perfect for this special issue on Hypertension in Asia 2021 in the *Journal of Clinical Hypertension,* to report on the latest developments from the continent which includes more than 60% of the global population. Contributions are expertly tailored according to the specific features of hypertension in the region, including greater salt sensitivity and nighttime hypertension—with many contributions from the Hypertension Cardiovascular Outcome Prevention and Evidence (HOPE) Asia Network. Importantly, contributions discuss unique local factors that could be targeted to prevent and manage hypertension, including dietary interventions while considering food cultures; Yoga, Tai chi, mindfulness‐based stress‐reduction programs, acupuncture, and Transcendental Meditation, as well as air pollution. Pharmacotherapy considerations include discussions of beta blockers (based on the Asia BP@Home study^3^), angiotensin receptor neprilysin inhibitor as a novel antihypertensive medication, and the “cardiovascular polypill,” which is still a few steps ahead of first‐line single pill combination hypertension therapy recommended by international guidelines, including the 2020 International Society of Hypertension (ISH) Guidelines.^4^

If there is one irreversible and beneficial consequence of COVID‐19, it is how innovatively digital technology is being used—over and beyond technological advances pre‐COVID. Not only is digital technology used for early surveillance, testing, quarantine, and contact tracing, but it is adopted and integrated into policy and health care.^5^ The potential unintended consequences of these developments may hold unprecedented value for health care in the future—embracing concepts such as big data, artificial intelligence, and machine learning, and employing telemonitoring, telemedicine and telecare also in other health spheres such as hypertension management. In the Hypertension in Asia 2021 issue, these facets are also neatly teased out, highlighting how emerging new technologies may offer even more possibilities in telecare, such as an instant interactive platform linking patients and health professionals. Asia is often highlighted as being ahead of the curve when it comes to technological advancements, as also mentioned by Whitelaw et al^5^ applauding how South Korea has integrated digital technology into government‐coordinated containment and mitigation processes during the COVID‐19 pandemic. The HOPE Asia Network is internationally recognized for its contributions in embracing out‐of‐office blood pressure. In this issue, the Network provides clear guidance on ambulatory blood pressure monitoring. With nighttime hypertension a common feature in Asia, the clinical significance of nighttime home blood pressure monitoring is also discussed as an alternative to traditional ambulatory blood pressure monitoring. Clearly, the increased risk of developing nighttime hypertension in Asian people (likely due to salt sensitivity and high salt intake) underscores precise management of nighttime blood pressures—with new technologies likely to facilitate better control in the coming years.

Additionally, this special issue includes comprehensive reviews on hypertension comorbidities and outcomes (from chronic kidney disease, stroke, obstructive sleep apnea, erectile dysfunction, obesity, mental health in the elderly, to arterial stiffness, and blood pressure variability) and diagnosis and risk assessment (specific risk assessment tools and screening for insulin resistance in Asian patients).

The 2020 ISH Global Hypertension Guidelines^4^ noted the importance to take ethnicity into account when managing hypertension as prevalence, treatment, and control rates vary according to ethnicity. These differences may be attributed to genetic differences, but environmental factors, such as lifestyle (eg, diet) and socioeconomic status, may alter health behaviors which may affect blood pressure. In this special issue, multiethnicity in Asia is appropriately discussed, as well as regional differences in home heart rates and ambulatory blood pressure profiles.

All possible efforts are required to lower blood pressure globally, including Asia. The close collaboration between different role players in Asia, including the contributions of the HOPE Asia Network, is showcased in detail in this special issue. This continued energy and dynamism toward a common goal of blood pressure control do not pass unnoticed, and along these trajectories, it is anticipated that significant improvements in hypertension management will be achieved in Asia. We all hope and also expect that in the next 1‐2 years, COVID‐19 will be conquered. May there be equal urgency globally to achieve similar results in hypertension.

## DISCLOSURES

AES has received speaker honoraria from Omron Healthcare, Servier, Takeda, Novartis and served a research advisory for Abbott.
